# Characteristics and outcomes of diabetes emergencies in nonagenarians admitted to ICU: a binational retrospective cohort study

**DOI:** 10.3389/fcdhc.2026.1769848

**Published:** 2026-03-11

**Authors:** Kyle Williams, Je Min Suh, Nattaya Raykateeraroj, Elif I. Ekinci, David Pilcher, Dong-Kyu Lee, Laurence Weinberg

**Affiliations:** 1 Department of Anaesthesia, Austin Health, Heidelberg, VIC, Australia; 2 Department of Anesthesiology, Faculty of Medicine Siriraj Hospital, Mahidol University, Bangkok, Thailand; 3 Department of Endocrinology, Austin Hospital, Melbourne, VIC, Australia; 4 Department of Medicine, Austin Health, Melbourne Medical School, The University of Melbourne, Melbourne, VIC, Australia; 5 Australian Centre for Accelerating Diabetes Innovations, Melbourne Medical School, The University of Melbourne, Melbourne, VIC, Australia; 6 The Alfred Intensive Care Academic Centre, Bayside Health, Melbourne, VIC, Australia; 7 The Australian and New Zealand Intensive Care Society (ANZICS) Centre for Outcome and Resource Evaluation, Melbourne, VIC, Australia; 8 The Australian and New Zealand Intensive Care Research Centre, School of Public Health and Preventive Medicine, Monash University, Melbourne, VIC, Australia; 9 Department of Anesthesiology and Pain Medicine, Dongguk University Ilsan Hospital, Goyang-si, Gyeonggi-do, Republic of Korea; 10 Department of Critical Care, University of Melbourne, Melbourne, VIC, Australia

**Keywords:** diabetes, diabetic ketoacidosis, elderly, hyperosmolar hyperglycemic state, intensive care, mortality, nonagenarian

## Abstract

**Introduction:**

Nonagenarian patients admitted to Intensive Care Units (ICU) are expected to rise with an ageing population. However, diagnosis-specific data is lacking in this cohort to guide clinical decisions. This includes diabetic emergencies, namely hyperosmolar hyperglycemic state (HHS) and diabetic ketoacidosis (DKA), which carry substantial morbidity and mortality. We sought to determine the incidence, clinical characteristics, and outcomes of nonagenarians admitted to ICUs across Australia and New Zealand (ANZ) with DKA and HHS to provide foundations for evidence-based prognostication and resource allocation.

**Methods:**

We conducted a binational multi-center retrospective analysis of nonagenarian patients admitted to ANZ ICUs with DKA or HHS between 2018-2024. Data was sourced from the Australian and New Zealand Intensive Care Society Centre for Outcome and Resource Evaluation. Our primary outcome was to determine the incidence of nonagenarians ICU admissions for diabetic emergencies. Baseline characteristics, physiological and biochemical data, length-of-stay (LOS) and mortality were compared between groups. Multivariable regression models were used to explore associations between diagnosis, complications, interventions, and ICU and hospital LOS. Cox proportional hazards models assessed effects of diagnosis on ICU and hospital LOS, and mortality.

**Results:**

19,078 nonagenarian patients were admitted to an ANZ ICU during the study period, and 86 (0.45%) were admitted with DKA (55 (64.0%)) or HHS (31 (36.0%)). Pre-admission diabetes related complications were more prevalent in HHS compared to DKA (100% vs 80%, P = 0.004), with no other significant differences in demographic, clinical and biochemical measures. DKA patients had significantly lower odds of developing AKI compared to HHS (adjusted OR 0.29, 95% CI 0.08 to 0.98; P = 0.046). ICU and hospital LOS did not differ significantly between groups. ICU mortality occurred in 1 (1.8%) DKA patient and 3 (9.7%) HHS patients, and in-hospital mortality in 7 (12.7%) and 6 (19.4%) patients, respectively. Survival over 48-months did not differ between groups (log-rank P = 0.790).

**Conclusion:**

Our findings suggest favourable outcomes can be achieved in suitable nonagenarians with reversible endocrine emergencies through ICU admission. We provide the first detailed description of this cohort, where future functional outcomes and quality of life assessment post-ICU admission could further inform triage decision-making.

## 1 Introduction

As life expectancy increases, the global population of nonagenarians in high-income countries is projected to exceed 30 million by 2030 (1). Consequently, the number of very elderly patients (≥ 90 years) admitted to intensive care units (ICU) is rising, driven by advancements in medical and surgical care now being offered to this age group (2, 3). This demographic shift presents complex challenges for intensive care medicine. Decisions regarding appropriateness of ICU admission in nonagenarians often involve consideration of multimorbidity, frailty, reduced physiological reserve and pre-existing treatment limitations (4). Clinical decision-making is further complicated by prognostic uncertainty, unknown implications on long-term quality of life, varying clinician attitudes toward ageing, and concerns of resource allocation (2, 5). Notably, recent studies suggest that chronological age alone is not an independent predictor of ICU mortality and therefore should not be the sole criterion guiding ICU admission decisions (4–8). This highlights the need for both diagnosis-specific and granular data on admission characteristics, morbidity, and mortality to inform ICU patient selection and treatment decisions in this highly comorbid and vulnerable group (9).

ICU mortality is significant among nonagenarians, with medical presentations associated with poorer short- and long-term outcomes than surgical admission (5, 10). This disparity highlights the importance of examining medical presentations that prompt ICU admission, an area underexplored in the very elderly. Among these are life-threatening diabetic emergencies, namely diabetic ketoacidosis (DKA) and hyperosmolar hyperglycemic state (HHS), which are associated with increased mortality in older patients (11–13). HHS has a mortality rate between 5-20%, compared with approximately 1% in DKA (14, 15). DKA is characterized by hyperglycemia, ketonemia, and metabolic acidosis, while HHS presents with severe hyperglycemia, hyperosmolarity and dehydration without significant acidosis or ketonemia. In general, DKA is more common in patients with type 1 diabetes (T1D), whereas HHS occurs more frequently in older patients with type 2 diabetes (T2D) (12, 14). Despite the clinical importance of these syndromes, no prior study has specifically examined ICU admissions for DKA or HHS among nonagenarians.

The present study aims to describe the incidence, clinical characteristics, and outcomes of nonagenarians admitted to ICUs across Australia and New Zealand with diabetic emergencies, providing a foundation for evidence-based prognostication and resource allocation in an ageing population.

## 2 Materials and methods

### 2.1 Study population & setting

We performed a binational multi-center retrospective analysis of prospectively collected data of patients admitted to Australian and New Zealand Intensive Care Units between 1 January 2018, and 21 December 2024. All study data was obtained from the Australian and New Zealand Intensive Care Society (ANZICS) Centre for Outcome and Resource Evaluation (CORE) Adult Patient Database (APD). Hospitalized adults ≥ 90 years admitted to the ICU for management of DKA or HHS were included in this study.

The Alfred Hospital Ethics Committee approved this study (project No. 253/24) and waived the requirement for informed consent because of the study’s retrospective nature and use of de-identified data. Data analysis commenced only after ethics approval had been obtained. The study was conducted in accordance with the Strengthening the Reporting of Observational Studies in Epidemiology (STROBE) guidelines (16).

### 2.2 Data collection

Demographic, clinical and physiological data were extracted from the ANZICS CORE APD. Demographic data included age, gender and source of admission. Clinical data included principal diagnosis, comorbidities, frailty defined by the Clinical Frailty Score (CFS) and illness severity at admission assessed by Acute Physiology and Chronic Health Evaluation III score (APACHE III). Physiological data included laboratory data, Glasgow Coma Scale (GSC) score, and temperature, all collected on ICU admission. Clinical outcomes extracted included development of acute kidney injury (AKI), delirium, or inotrope requirement, ICU and hospital length of stay (LOS) as well as mortality in-ICU, in-hospital, and up to 48-months post ICU discharge.

### 2.3 Outcome measures

Given the exploratory and observational design of this study, our primary objectives were to describe the incidence of ICU admission for management of DKA or HHS among nonagenarian patients across Australia and New Zealand. The secondary objectives were to determine baseline patient characteristics including frailty, comorbidities, APACHE III score, physiological parameters on ICU admission, ICU and hospital length of stay, and clinical outcomes including acute kidney injury, delirium, inotrope requirement and mortality.

We also explored association between the HHS and DKA groups with respect to key physiological measures, ICU and hospital length of stay, and clinical outcomes including AKI, delirium, inotrope requirement and survival up to 48-months post discharge. Cumulative survival over time was assessed using Kaplan–Meier curves.

### 2.4 Definitions

Data extraction and variable definitions adhered to the specifications provided in the ANZICS CORE APD Data Dictionary to ensure standardization across contributing sites. Diabetic ketoacidosis was defined as a serum glucose concentration exceeding 13.9 mmol/L, arterial pH below 7.30, serum bicarbonate less than 18 mmol/L, and the presence of ketonemia or ketonuria, consistent with the American Diabetes Association 2024 Consensus Report (14). Hyperosmolar hyperglycemic state was defined as a serum glucose concentration exceeding 33 mmol/L, effective serum osmolality greater than 320 mOsm/kg, minimal or absent ketones, and an arterial pH above 7.30. AKI was defined according to the Kidney Disease: Improving Global Outcomes (KDIGO) criteria (17), based on changes in serum creatinine or urine output recorded during the ICU stay.

Management of DKA and HHS was guided by local institutional protocols aligned with nationally accepted evidence-based guidelines, focusing on timely fluid resuscitation, intravenous insulin therapy, careful electrolyte replacement, and identification and treatment of precipitating causes. Clinical care variations reflected site-specific practices, but all participating ICUs adhered to standardized approaches consistent with contemporary critical care management across Australia and New Zealand, as outlined by ANZICS CORE.

### 2.5 Statistical analysis

All statistical analyses were conducted in R software, version 4.4.3 (R Foundation for Statistical Computing, Vienna, Austria). Baseline demographic, clinical, and biochemical characteristics were compared between groups. Continuous variables were assessed for normality with the Shapiro–Wilk test, and visual inspection of histograms and Q–Q plots. Because most continuous variables were non-normally distributed, they are summarized as medians with interquartile ranges and were compared with the Mann–Whitney U test. Categorical variables are presented as frequencies with percentages and were compared with Pearson’s χ² test or Fisher’s exact test, as appropriate.

Univariable and multivariable logistic and linear regression models were used for binary and continuous outcomes, respectively, with multivariable models adjusted for age, sex, frailty, illness severity (APACHE III score), neurological status (GCS), and the complete comorbidity profile available in the dataset. Log-transformation was applied where appropriate for continuous variables with right-skewed distribution prior to analysis with linear regression. Cox Proportional Hazard Regression was used to estimate hazard ratios (HRs) comparing ICU, hospital and post-discharge mortality between groups. Time-to-event outcomes, including mortality up to 48-months, was assessed using Kaplan-Meier survival curves with log-rank tests to determine significance in differences. All *p* values < 0.05 were considered statistically significant.

Results from logistic regression are presented as odds ratios (ORs), from linear regression as beta coefficients (β), and from Cox models as hazard ratios (HRs), each with 95% confidence intervals. For log-transformed outcomes prior to analysis, beta coefficients were exponentiated (Expβ) to facilitate interpretation of the original scale. Model assumptions were tested and met for all regression analyses. Missing data were handled using complete case analysis for logistic regression, and no imputation was performed for missing data for linear regression (see **Supplementary Table 1**).

## 3 Results

### 3.1 Primary outcome: prevalence of nonagenarian ICU admissions for DKA and HHS

Over the study period, 19,078 nonagenarian patients were admitted to an Australian or New Zealand ICU, of which 86 (0.45%) were admitted for either DKA or HSS and included in our analysis. The study population included 55 patients (64.0%) with DKA and 31 patients (36.0%) with HHS over the seven-year inclusion period (**Table 1**). While the database included both nonagenarians and centenarians, no centenarian patients were admitted for either DKA or HHS during the study period.

**Table 1:** Baseline characteristics, physiological measures, and clinical outcomes of nonagenarian patients admitted to ICU with diabetic ketoacidosis (DKA) versus hyperosmolar hyperglycemic state (HHS).

Variable	DKA (n=55)	HHS (n=31)	P value
Male sex	22 (40)	14 (45.2)	0.812
Chronic respiratory disease	3 (5.5)	3 (9.7)	0.766
Chronic cardiovascular disease	13 (23.6)	9 (29.0)	0.769
Chronic liver disease	0	0	>0.999
Chronic renal disease	5 (9.1)	3 (9.7)	1.000
Diabetes complicated by nephropathy, retinopathy, neuropathy or macrovascular disease	44 (80)	31 (100)	0.004*
Acute kidney injury	31 (56.4)	25 (80.6)	0.042*
Delirium during ICU stay	20 (36.4)	13 (41.9)	0.780
Inotrope requirement	5 (9.1)	2 (6.5)	0.985
ICU mortality	1 (1.8)	3 (9.7)	0.259
Hospital mortality	7 (12.7)	6 (19.4)	0.610
Post-discharge mortality	27 (49.1)	19 (61.3)	0.388
1-month mortality	6 (10.9)	6 (19.4)	0.242
6-month mortality	14 (25.5)	14 (45.2)	0.102
12-month mortality	20 (36.4)	16 (51.6)	0.251
24-month mortality	26 (47.3)	20 (64.5)	0.189
48-month mortality	34 (61.8)	24 (77.4)	0.214
Age (years)	91.5 [90.6–93.7]	92.2 [90.8–94.2]	0.506
Frailty score	5 [3–5.7]	6 [4–7]	0.443
Serum lactate (mmol/L)	2.1 [1.8–3.0]	2.7 [2.0–4.0]	0.197
Glasgow Coma Scale score	14 [11–15]	14 [11–15]	0.084
Temperature (°C)	36.0 [35.5–36.3]	36.0 [35.7–36.3]	0.586
Arterial pH	7.4 [7.3–7.4]	7.4 [7.3–7.4]	0.386
Serum urea (mmol/L)	14.9 [9.7–19.2]	15.6 [12.6–20.1]	0.182
Serum albumin (g/L)	28 [24–32]	29 [23.5–31]	0.534
Blood glucose (mmol/L)	20.0 [14.7–30.2]	24.9 [15.7–27.5]	0.693
Lowest MAP (mmHg)	65 [59.7–66]	65 [59–70]	0.081
Highest bicarbonate (mmol/L)	20 [19–25]	24 [21–25.6]	0.074
Lowest bicarbonate (mmol/L)	18 [17–25]	22 [18–25]	0.386
Highest creatinine (*u*mol/L)	98 [81–156.5]	127 [99.5–161.5]	0.179
Lowest haemoglobin (g/L)	111.4 [81.9–118]	99.5 [81.9–162]	0.179
APACHE III score	70 [61–81]	75 [67.5–87.5]	0.088
Hospital length of stay (days)	11.8 [6.5–23.2]	8.9 [6.5–17.4]	0.079
ICU length of stay (days)	2.0 [1.0–3.1]	2.3 [1.1–3.4]	0.643

ICU, intensive care unit; MAP, mean arterial pressure; APACHE III, Acute Physiology and Chronic Health Evaluation III score. Data are presented as number (percentage) for categorical variables and median [interquartile range] for continuous variables. Comparisons between groups were performed using the chi-squared test or Fisher's exact test for categorical variables, and Wilcoxon rank-sum test for continuous variables. P value <0.05*.

### 3.2 Clinical characteristics and physiological measures

Among this cohort (**Table 1**), the median (IQR) age was similar between DKA and HHS groups (91.5 years [90.6–93.7] vs 92.2 [90.8–94.2], P = 0.506), and sex distribution was comparable, with males comprising 40% of the DKA group 45.2% of the HHS group (P = 0.812). Diabetes related complications i.e., nephropathy, retinopathy, neuropathy or macrovascular disease were more prevalent in patients with HHS compared to DKA (100% vs 80%, P = 0.004), while other comorbidities, including cardiovascular, respiratory, renal and liver disease, were broadly similar between groups.

Patients with DKA had slightly lower Clinical Frailty Score (CFS) compared to those with HHS (median 5 [3–5.7] vs 6 [4–7], P = 0.443), though both groups demonstrated moderate frailty on admission, reflecting the overall vulnerability of this ICU population. Illness severity on ICU admission was similar between groups, with a median APACHE III score of 70 [60–81] in the DKA group and 75 [67.5–87.5] in the HHS group (P = 0.088).

Vital signs on admission, including temperature, GCS score, and mean arterial pressure were similar between groups. Nonagenarians with DKA had lower bicarbonate levels compared with HHS (18 [17–25] vs 22 [18–25] mmol/L), however this difference was not statistically significant (P = 0.386). Further, there were no significant differences observed in other laboratory values, including blood glucose, arterial pH, lactate, creatinine, urea, albumin, and hemoglobin. A full summary of demographic, clinical and biochemical variables is presented in **Table 1**.

### 3.3 Clinical outcomes

Clinical outcomes are summarised in **Table 1**. Common clinical outcomes included delirium (36.4% in DKA vs 41.9% in HHS, P = 0.780) and requirement for inotrope support (9.1% vs 6.5%, P = 0.985), with no significant difference between groups. Notably, AKI occurred significantly more often in nonagenarians with HHS compared to DKA (80.6% vs 56.4%, P = 0.042).

ICU and hospital length of stay (LOS) did not differ significantly between groups. Median ICU stay was 2.0 days [1.0–3.1] in DKA compared to 2.3 [1.1–3.4] for HHS (P = 0.643), while hospital stay was longer in DKA patients (11.8 days [6.5–23.2]) compared to HHS patients (8.9 days [6.5–17.4], P = 0.079). ICU mortality was low across the cohort, with 1 death (1.8%) in the DKA group, and 3 (9.7%) in the HHS group (P = 0.259). Similarly, hospital mortality was comparable between groups (7 deaths (12.7%) in DKA vs 6 (19.4%) in HHS, P = 0.610).

### 3.4 Association between admission diagnosis and ICU complications

In unadjusted analyses, patients admitted with DKA had significantly lower odds of developing AKI compared with those presenting with HHS (OR 0.31, 95% CI 0.11 to 0.88; P = 0.027), and this association remained significant in the fully adjusted model (adjusted OR 0.29, 95% CI 0.08 to 0.98; P = 0.046) (**Table 2**). Higher illness severity was independently associated with AKI, with each unit increase in APACHE III score corresponding to higher odds of AKI (adjusted OR 1.07, 95% CI 1.02 to 1.12; P = 0.005). GCS, frailty, sex, and age were not significant predictors of AKI.

**Table 2:** Association between ICU admission diagnosis and complications.

Outcome	Variable	Univariate OR (95% CI)	P value	Multivariate OR (95% CI)	P value
Acute kidney injury	DKA vs HHS	0.310 (0.110–0.876)	0.027	0.286 (0.084–0.978)	0.046
	Apache III score	1.040 (1.010–1.080)	0.007	1.070 (1.020–1.120)	0.005
	Glasgow Coma Scale score	0.976 (0.801–1.190)	0.813	1.310 (0.936–1.830)	0.115
	Frailty	1.000 (0.774–1.300)	0.993	0.949 (0.657–1.370)	0.781
	Male sex	1.120 (0.456–2.770)	0.798	0.634 (0.197–2.040)	0.445
	Age	0.964 (0.812–1.140)	0.669	0.989 (0.803–1.220)	0.921
Delirium	DKA vs HHS	0.791 (0.322–1.950)	0.610	0.845 (0.286–2.500)	0.761
	Apache III score	1.030 (1.010–1.060)	0.018	1.020 (0.987–1.060)	0.202
	Glasgow Coma Scale score	0.746 (0.590–0.944)	0.015	0.794 (0.570–1.110)	0.173
	Frailty	1.070 (0.829–1.380)	0.604	0.871 (0.617–1.230)	0.431
	Male sex	0.848 (0.350–2.050)	0.715	0.889 (0.305–2.590)	0.830
	Age	0.840 (0.690–1.020)	0.083	0.811 (0.645–1.020)	0.074
Inotrope requirement	DKA vs HHS	1.450 (0.264–7.960)	0.669	2.190 (0.274–17.500)	0.460
	Apache III score	1.020 (0.979–1.060)	0.393	0.994 (0.942–1.050)	0.829
	Glasgow Coma Scale score	0.816 (0.638–1.040)	0.105	0.671 (0.396–1.140)	0.140
	Frailty	1.410 (0.843–2.360)	0.190	1.160 (0.627–2.140)	0.638
	Male sex	0.529 (0.097–2.900)	0.463	0.734 (0.099–5.420)	0.762
	Age	0.970 (0.707–1.330)	0.849	0.869 (0.550–1.380)	0.550

OR, odds ratio; CI, confidence interval; DKA, diabetic ketoacidosis; HHS, hyperosmolar hyperglycaemic state. Univariate models present the unadjusted association between each predictor and the outcome. Multivariate models are fully adjusted for age, sex, frailty, illness severity (APACHE III score), neurological status (Glasgow Coma Scale), and the complete comorbidity profile available in the dataset. These comorbidities include chronic respiratory disease, chronic cardiovascular disease, chronic liver disease, chronic renal disease, immunodeficiency, receipt of immunosuppressive therapy, hepatic failure, lymphoma, metastatic malignancy, leukaemia, general immunosuppression, and cirrhosis. All comorbidity variables were included simultaneously in the multivariable logistic regression models to ensure appropriate adjustment for baseline disease burden.

For delirium (**Table 2**), there was no significant difference between DKA and HHS in univariate (OR 0.79, 95% CI 0.32 to 1.95; P = 0.610) or multivariable models (adjusted OR 0.85, 95% CI 0.29 to 2.50; P = 0.761). Although APACHE III score (OR 1.03, 95% CI 1.01 to 1.06; P = 0.018) and lower GCS (OR 0.75, 95% CI 0.59 to 0.94; P = 0.015) were associated with delirium in univariate analyses, both effects attenuated following multivariable adjustment (adjusted OR 1.02, 95% CI 0.99 to 1.06; P = 0.202 and adjusted OR 0.79, 95% CI 0.57 to 1.11; P = 0.173, respectively). Age, frailty, and sex were not independently associated with delirium.

There were no significant predictors of inotrope requirement (**Table 2**), with DKA showing no association in either unadjusted (OR 1.45, 95% CI 0.26 to 7.96; P = 0.669) or adjusted analyses (adjusted OR 2.19, 95% CI 0.27 to 17.50; P = 0.460). APACHE III score (adjusted OR 0.99, 95% CI 0.94 to 1.05; P = 0.829), GCS (adjusted OR 0.67, 95% CI 0.40 to 1.14; P = 0.140), frailty (adjusted OR 1.16, 95% CI 0.63 to 2.14; P = 0.638), sex (adjusted OR 0.73, 95% CI 0.10 to 5.42; P = 0.762), and age (adjusted OR 0.87, 95% CI 0.55 to 1.38; P = 0.550) were also not significant predictors, although interpretation was limited by wide confidence intervals due to low event counts.

### 3.5 Association between admission diagnosis and ICU and hospital length of stay

Diagnosis of DKA was not associated with ICU LOS (**Table 3**) (ratio 1.00, 95% CI 0.72 to 1.40; P = 0.999). In contrast, higher illness severity was independently associated with longer ICU stay, with each unit increase in APACHE III score corresponding to a 1.8% relative increase in ICU LOS (ratio 1.018, 95% CI 1.01 to 1.03; P = 0.002). GCS, frailty, sex, age, and all comorbidity variables showed no significant associations with ICU LOS. For hospital LOS, DKA was not significantly associated with duration of stay (ratio 1.36, 95% CI 0.92 to 2.02; P = 0.127). A higher GCS was the only significant predictor, with each point associated with a 12.9% longer hospital stay (ratio 1.129, 95% CI 1.01 to 1.26; P = 0.030). APACHE III score, frailty, age, sex, and comorbidities were not associated with hospital LOS. No comorbidity variable demonstrated a significant independent effect on either ICU or hospital length of stay after adjustment for demographic, physiological, and illness severity factors.

**Table 3:** Association between ICU admission diagnosis and length of stay.

Outcome	Variable	Ratio (95% CI)	P value
ICU length of stay	DKA vs HHS	1.000 (0.717–1.396)	0.999
	Apache III score	1.018 (1.007–1.029)	0.002
	Glasgow Coma Scale score	1.048 (0.956–1.148)	0.314
	Frailty	0.964 (0.868–1.071)	0.486
	Male sex	0.830 (0.601–1.146)	0.254
	Age	0.996 (0.936–1.059)	0.886
	Chronic respiratory disease	1.299 (0.681–2.478)	0.422
	Chronic cardiovascular disease	1.106 (0.708–1.729)	0.654
	Chronic renal disease	0.563 (0.274–1.155)	0.115
	Immunodeficiency	1.114 (0.251–4.954)	0.885
	Hepatic failure	2.164 (0.444–10.536)	0.334
	Metastatic cancer	0.658 (0.338–1.282)	0.215
	Leukaemia	0.692 (0.143–3.346)	0.643
Hospital length of stay	DKA vs HHS	1.360 (0.915–2.023)	0.127
	Apache III score	1.009 (0.996–1.022)	0.166
	Glasgow Coma Scale score	1.129 (1.012–1.259)	0.030
	Frailty	1.017 (0.897–1.153)	0.791
	Male sex	1.177 (0.801–1.728)	0.401
	Age	0.981 (0.912–1.055)	0.601
	Chronic respiratory disease	0.747 (0.346–1.613)	0.453
	Chronic cardiovascular disease	1.114 (0.654–1.897)	0.687
	Chronic renal disease	1.078 (0.458–2.537)	0.862
	Immunodeficiency	0.922 (0.156–5.450)	0.927
	Hepatic failure	0.432 (0.066–2.849)	0.378
	Metastatic cancer	0.576 (0.260–1.275)	0.171
	Leukaemia	0.600 (0.092–3.925)	0.590

DKA, diabetic ketoacidosis; HHS, hyperosmolar hyperglycaemic state. Ratios represent the exponentiated coefficients from log-transformed linear regression models, reflecting multiplicative effects on ICU or hospital length of stay. A ratio greater than 1 indicates longer stay, whereas a ratio below 1 indicates shorter stay. Models were adjusted for illness severity, neurological status, frailty, demographic factors, and comorbidities including chronic respiratory, cardiovascular, liver, and renal disease; immunodeficiency; immunosuppressive conditions; hepatic failure; lymphoma; metastatic cancer; and leukaemia.

### 3.6 Association between admission diagnosis and mortality

DKA was not significantly associated with hospital mortality in unadjusted (HR 0.74, 95% CI 0.25 to 2.21; P = 0.588) or multivariable analyses (adjusted HR 0.61, 95% CI 0.17 to 2.25; P = 0.459) (**Table 4**). Illness severity was not independently associated with hospital mortality after adjustment (adjusted HR 1.00, 95% CI 0.96 to 1.04; P = 0.826). A lower GCS remained the only significant predictor of hospital mortality, with each point decrease associated with higher mortality risk (adjusted HR 0.71, 95% CI 0.51 to 0.997; P = 0.048). Frailty, sex, and age showed no significant associations with hospital mortality following adjustment. For ICU mortality, there was no significant difference between DKA and HHS in either univariate (HR 0.24, 95% CI 0.03 to 2.30; P = 0.216) or multivariable models (adjusted HR 0.24, 95% CI 0.01 to 6.44; P = 0.392). APACHE III score and frailty were not significantly associated with ICU mortality after adjustment (adjusted HR 0.99, 95% CI 0.89 to 1.10; P = 0.872 and adjusted HR 0.42, 95% CI 0.11 to 1.63; P = 0.210, respectively). GCS demonstrated an association in univariate analysis (HR 0.77, 95% CI 0.62 to 0.96; P = 0.018), but the effect attenuated in the adjusted model (adjusted HR 0.55, 95% CI 0.25 to 1.24; P = 0.152). Sex and age were not independent predictors of ICU mortality. Precision for ICU mortality estimates was limited by the small number of events.

**Table 4:** Cox proportional hazards regression of ICU and hospital mortality.

Outcome	Variable	Univariate HR (95% CI)	P value	Multivariate HR (95% CI)	P value
Hospital Mortality	DKA vs HHS	0.740 (0.248–2.210)	0.588	0.611 (0.166–2.250)	0.459
	Apache III score	1.020 (0.998–1.050)	0.074	0.996 (0.957–1.040)	0.826
	Glasgow Coma Scale score	0.791 (0.685–0.913)	0.001	0.713 (0.510–0.997)	0.048
	Frailty	1.140 (0.811–1.600)	0.454	0.907 (0.552–1.490)	0.700
	Male sex	0.537 (0.165–1.740)	0.300	0.465 (0.119–1.820)	0.270
	Age	0.952 (0.764–1.190)	0.665	0.814 (0.599–1.110)	0.191
ICU Mortality	DKA vs HHS	0.240 (0.025–2.300)	0.216	0.236 (0.009–6.440)	0.392
	Apache III score	1.030 (0.985–1.070)	0.216	0.991 (0.891–1.100)	0.872
	Glasgow Coma Scale score	0.772 (0.624–0.956)	0.018	0.552 (0.245–1.240)	0.152
	Frailty	0.835 (0.470–1.490)	0.540	0.419 (0.108–1.630)	0.210
	Male sex	0.408 (0.043–3.930)	0.438	0.606 (0.014–25.700)	0.793
	Age	0.966 (0.648–1.440)	0.864	1.150 (0.556–2.360)	0.712

HR, hazard ratio; CI, confidence interval; DKA, diabetic ketoacidosis; HHS, hyperosmolar hyperglycaemic state. Hazard ratios and 95% confidence intervals are presented for both univariate and multivariate Cox proportional hazards models using time from ICU admission to death as the time-to-event variable. Multivariate models were adjusted for illness severity (APACHE III score), neurological status (Glasgow Coma Scale), frailty, sex, age, and comorbidities including chronic respiratory, cardiovascular, liver, and renal disease; immunodeficiency; immunosuppressive therapy; hepatic failure; lymphoma; metastatic cancer; leukaemia; general immunosuppression; and cirrhosis.

**Figure 1:**
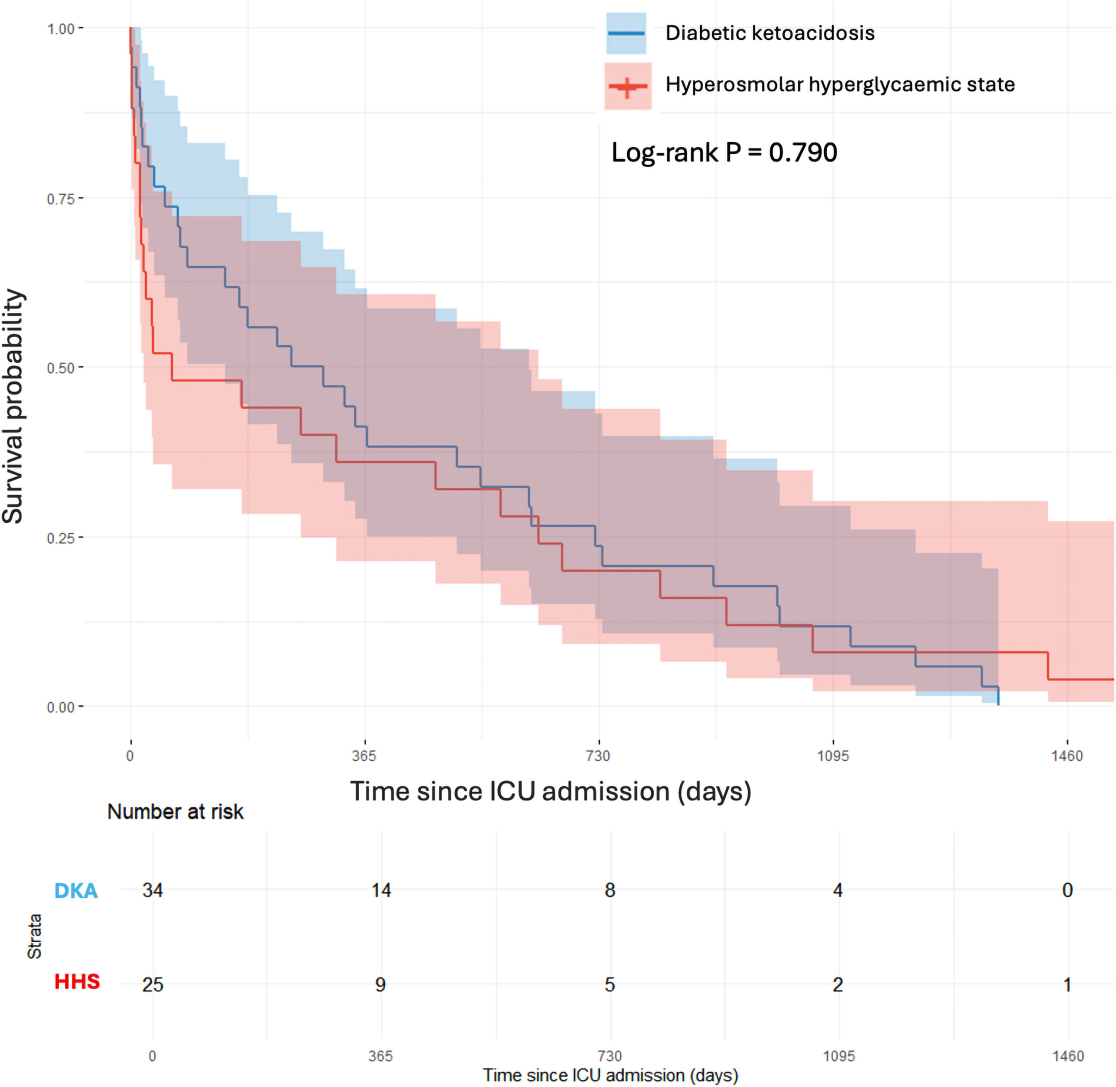
Kaplan-Meier Curve comparing 48-month survival between patients admitted with HHS and those with DKA. Shaded regions represent 95% confidence intervals. Risk tables below the graph display the number of patients at risk, censored, and the number of observed deaths over time in each group. Proportional hazards assumption was assessed using scaled Schoenfeld residuals and was not violated, supporting the use of Cox proportional hazards modelling.

Survival over 48 months did not differ significantly between patients with DKA and those with HHS, with largely overlapping confidence bands throughout follow-up (log-rank P = 0.790) (**Figure 1**). Both groups demonstrated similar long-term mortality trajectories, and no divergence in survival curves was observed over time.

## 4 Discussion

### 4.1 Key findings

We performed a multi-centre binational retrospective study to investigate the characteristics and outcomes of nonagenarians admitted to an Australian or New Zealand ICU with a primary diagnosis of DKA or HHS. We found that ICU mortality was low, affecting 1.8% of patients with DKA and 9.7% with HHS. In-hospital mortality occurred in 12.7% and 19.4% of patients, respectively. Majority of this population were mild to moderately frail on admission, with a high prevalence of diabetes and cardiovascular disease. We found that delirium was the most common complication across both groups, while AKI occurred significantly more in those admitted for HHS. Admission diagnosis was not an independent predictor of long-term mortality, with 25% of DKA and 29% of HHS patients alive after 1-year.

### 4.2 Our findings in the context of existing literature

This is the first study to specifically characterise outcomes of ICU admission for DKA and HHS in nonagenarian patients. There is a dearth of existing literature on diabetic emergencies in the elderly. A recent scoping review by *Suh* et al. (2025) reported nonagenarian ICU mortality ranges from 35-60%, with median hospital mortality of 25.55%, and higher in medical admissions. In our cohort, ICU and hospital mortality were substantially lower than reported in the broader nonagenarian population, supporting the decision for admission. Further, *Kitisin* et al. (2025) demonstrate comparable mortality rates to younger patients when adjusted for illness severity (18). Together, our findings alongside the current literature suggests that favourable outcomes are achievable in well-selected patients with a reversible condition and supports the emphasis shared by clinicians on incorporating frailty, functional status and illness severity, rather than age alone, in admission decision making (2, 9, 19).

### 4.3 Predictors of mortality

Delirium was the most common complication in our cohort affecting over a third of all patients, consistent with the high incidence reported amongst critically ill patients admitted to ICU (20). This underscores its significance as a frequent complication which carries prognostic implications. Acute delirium has been associated with increased ICU and hospital mortality and prolonging length of stay, with episodes in the nonagenarian population likely having greater consequences due to their pre-existing frailty and susceptibility to deconditioning (21–23). These risks reinforce the need for early recognition and proactive prevention.

Although HHS was associated with greater biochemical disturbance and higher rates AKI, this did not translate into increased adjusted mortality compared with DKA. The predominance of AKI in HHS likely reflects the greater severity of dehydration and hyperosmolarity, further increasing vulnerability to renal injury in older patients (14, 24, 25). Although we did not specifically examine the association between AKI and mortality, prior studies in nonagenarians demonstrate AKI on admission is a predictor of in-hospital mortality (26). Even mild AKI increases risk, while severe AKI confers a six-fold increase in mortality (27). Beyond mortality, AKI in nonagenarians is linked to longer ICU and overall length of stay, vasopressor use and mechanical ventilation (27, 28). From our findings, APACHE III and CFS did not differ between DKA and HHS patients. Nonetheless, these and other illness severity measures and frailty scores are established predictors of mortality in elderly patients, and are used to support triage decisions (3, 7, 9, 18, 29, 30).

As ICU admission among very elderly patients rise, consideration of outcomes beyond survival is essential (8, 9). This includes discharge destination physical and cognitive recovery, and quality of life (5, 19). We evaluated long-term mortality up to four-years post-discharge, however, the trajectory of functional recovery remains unclear, underscoring the need for standardised reporting that encompasses patient-reported outcome measures (9).

### 4.4 Study implications

In nonagenarians admitted to ICU for management of HHS and DKA, ICU and hospital mortality was relatively low, suggesting favourable outcomes following a period of dedicated ICU care for these reversible endocrine emergencies. While ICU admission is often appropriate for severe presentations, disposition alternatives outside the ICU such as a high-dependency unit or ward-based setting for selected patients with uncomplicated mild-moderate DKA may also be considered (14). In such cases, there is potential to reduce laboratory testing and hospitalisation costs associated with ICU admission, and to mitigate strain on ICU capacity. However, this is dependent on institutional resources including suitable nursing allocation ratios, appropriate close monitoring, and clear escalation pathways (31). Therefore, early re-triage following stabilisation is important in supporting the smooth and timely transition of step-down care, while also facilitating efficient resource use and safe continuity of treatment (4).

Our findings reinforce the holistic view that suitability for ICU should be guided by frailty, illness severity, and patient preferences. Although nonagenarians represent a minority of ICU patients, they generally receive similar life-sustaining treatments to the octogenarian population, with comparable mortality (32, 33). Future studies that look beyond survival and hospital discharge are warranted to capture the impact on quality of life and functional recovery, to enable realistic discussions with patients and families.

### 4.5 Strengths and limitations

Our study has several notable strengths. The use of prospectively and routinely collected data from the bi−national ANZICS Adult Patient Database (APD) ensured rigorous data capture, predefined variable definitions, and high internal validity across more than 180 ICUs in Australia and New Zealand (33, 34). The registry’s standardised data collection processes, maintained through continuous quality auditing, enable benchmarking and minimise misclassification and reporting bias. Inclusion of longitudinal follow−up data for up to four years allowed for the evaluation of both short− and medium−term mortality beyond the index ICU admission. To our knowledge, this is the first detailed analysis focusing on nonagenarians admitted to the ICU with diabetic emergencies, incorporating demographic, physiological, and clinical outcome data. These findings help characterise an understudied population and may inform triage and resource allocation decisions in ageing societies.

However, several limitations merit consideration. First, the relatively small number of nonagenarians admitted with DKA or HHS limited statistical power and reflects the rarity of such admissions rather than a methodological shortcoming. Second, registry data only permit the recording of a single primary diagnosis, which may result in underrepresentation of patients whose precipitating cause (for example, sepsis, insulin omission, or myocardial infarction) was coded as the principal diagnosis rather than DKA or HHS. Consequently, the underlying cause of hyperglycaemic crisis could not always be ascertained. Third, specific treatment−related data, including timing and composition of intravenous fluids, insulin administration, and electrolyte replacement, were not captured, restricting analysis to higher−level interventions. Similarly, laboratory measures such as serum or urinary ketones are not systematically recorded in the ANZICS APD, and ICU admission often follows partial initial resuscitation; thus, the defining metabolic differences between DKA and HHS at presentation may have been attenuated (14). Finally, post−discharge quality−of−life measures, functional outcomes, and patient−reported indicators were unavailable, precluding assessment of longer−term recovery and survivorship trajectories. As a result, while objective morbidity and mortality can be robustly described, patient−centred outcomes remain an important area for future registry development.

## 5 Conclusion

This binational retrospective cohort study provides the first detailed description of the incidence, clinical characteristics and outcomes of nonagenarians admitted to an Australian or New Zealand ICU for management of DKA or HHS. Our findings demonstrate ICU and hospital mortality were lower than reported in broader nonagenarian ICU populations, and provided insight into long-term survival, indicating that carefully selected nonagenarians with reversible endocrine emergencies can achieve favourable outcomes. Future focus towards understanding the functional outcomes and quality of life of nonagenarian’s post-discharge following an ICU admission would further inform triage decision-making in this population.

## Data Availability

The data analyzed in this study is subject to the following licenses/restrictions: The full de-identified dataset is available from the corresponding author upon reasonable request. Requests to access these datasets should be directed to laurence.weinberg@austin.org.au.
