# Supplementary material for: Characteristics and outcomes of diabetes emergencies in nonagenarians admitted to ICU: a binational retrospective cohort study
Source: Front Clin Diabetes Healthc. 2026 Mar 11;7:1769848. doi: 10.3389/fcdhc.2026.1769848 (PMC13013046; doi:10.3389/fcdhc.2026.1769848)
Supplement: Supplementary file 1 [file Table1.docx]

**Supplementary File Table 1.** Missing data

| **Missing Variable** | **DKA (n=55)** | **HHS (n=31)** |
| --- | --- | --- |
| **Baseline characteristics**  Age  Gender  Hospital source  ICU source  Private / public | 0  0  0  0  0 | 0  0  0  0  0 |
| **Baseline health measures**  Frailty  Chronic respiratory disease  Chronic cardiovascular Disease  Chronic liver disease  Chronic renal disease  Diabetes  APACHE III Score | 0  0  0  0  0  9 (16.4)  0 | 0  0  0  0  0  0  0 |
| **ICU Admission Clinical Variables**  Lactate  Glascow Coma Scale score  Temperature  pH  Urea  Albumin  Glucose  Mean arterial pressure  HCO_3_: High  HCO_3_: Low  Creatinine  Haemoglobin: Low | 0  0  0  1 (1.8)  0  0  0  0  0  2 (3.6)  0  0  0 | 0  0  0  0  0  0  0  1 (3.0)  0  0  0  0  0 |
| **Admission Outcome**  Acute kidney injury  Delirium  Inotrope usage  Hospital mortality  ICU mortality  Post discharge mortality  30-day mortality  6-month mortality  12-month mortality  24-month mortality  48-month mortality  ICU length of stay  Hospital length of stay | 0  0  0  0  0  0  0  0  0  0  0  0  0 | 0  0  0  0  0  0  0  0  0  0  0  0  0 |
| *Abbreviations: DKA: Diabetic Ketoacidosis; HHS: Hyperosmolar Hyperglycaemic State; ICU: intensive care unit; HCO₃: bicarbonate.* Data are reported as number (percentage) of patients with missing information. Baseline characteristics refer to demographic factors and admission sources. Baseline health measures encompass pre-existing comorbidities and severity of illness scores. ICU admission clinical variables comprise physiological and biochemical parameters collected at the time of ICU admission. Admission outcomes include in-hospital complications, mortality at multiple time points, and hospital and ICU lengths of stay. | | |
